# Diagnostic Utility of Total IgE in Foods, Inhalant, and Multiple Allergies in Saudi Arabia

**DOI:** 10.1155/2016/1058632

**Published:** 2016-05-25

**Authors:** Jamil A. Al-Mughales

**Affiliations:** ^1^Diagnostic Immunology Division, Department of Clinical Laboratory Medicine, King Abdulaziz University Hospital, P.O. Box 80215, Jeddah 21589, Saudi Arabia; ^2^Department of Medical Microbiology and Immunology, Faculty of Medicine, King Abdulaziz University, P.O. Box 80215, Jeddah 21589, Saudi Arabia

## Abstract

*Objective.* To assess the diagnostic significance of total IgE in foods, inhalant, and multiple allergies.* Methods.* Retrospective review of the laboratory records of patients who presented with clinical suspicion of food or inhalant allergy between January 2013 and December 2014. Total IgE level was defined as positive for a value >195 kU/L; and diagnosis was confirmed by the detection of specific IgE (golden standard) for at least one food or inhalant allergen and at least two allergens in multiple allergies.* Results.* A total of 1893 (male ratio = 0.68, mean age = 39.0 ± 19.2 years) patients were included. Total IgE had comparable sensitivity (55.8% versus 59.6%) and specificity (83.9% versus 84.4%) in food versus inhalant allergy, respectively, but a superior PPV in inhalant allergy (79.1% versus 54.4%). ROC curve analysis showed a better diagnostic value in inhalant allergies (AUC = 0.817 (95% CI = 0.796–0.837) versus 0.770 (95% CI = 0.707–0.833)). In multiple allergies, total IgE had a relatively good sensitivity (78.6%), while negative IgE testing (<195 kU/L) predicted the absence of multiple allergies with 91.5% certitude.* Conclusion.* Total IgE assay is not efficient as a diagnostic test for foods, inhalant, or multiple allergies. The best strategy should refer to specific IgE testing guided by a comprehensive atopic history.

## 1. Introduction

Immunoglobulin E (IgE) predominantly mediates immunity and immune responses against parasitic infections, but it is also an essential component of type I hypersensitivity reaction [1], which can cause anaphylaxis, asthma, atopic dermatitis, and allergic rhinitis [2, 3]. Inhalant and food allergies are induced and regulated by IgE and can be present in children and adults with frequent or chronic upper respiratory inflammatory episodes that are often misdiagnosed as viral infections [4]. Allergy is increasingly common worldwide: 20%–25% of adults reportedly have an allergy-based respiratory disease [5], and up to 40% of children in western countries may be affected [6–8]. Children who are genetically prone to atopy commonly present with eczema up to the age of 3 years, after which asthma and rhinitis develop as the next stage of the “atopic march” [7]. The most effective treatment is prompt diagnosis followed by the identification of specific causative allergen(s) [9].

The gold standard for the detection of specific allergens is the ImmunoCAP® immunoassay, but this method can be costly and requires specialist equipment and skill. Many immunologists therefore initially assess the total IgE levels in patients with suspected allergies, despite the reported low negative predictive value of this assay [10–13]. Currently, the measurement of total IgE is recommended only as a supplemental diagnostic measure for the diagnosis of allergic asthma [14]. However, this investigation is widely used by clinicians in the Middle East, including those in Saudi Arabia, even though the efficacy and cost-effectiveness of assessing total IgE remain unclear.

This study aimed to assess the predictive value of total IgE in a group of patients with suspected allergies in Saudi Arabia, in order to determine whether this test is useful as a diagnostic tool in this population. Moreover, the predictive value of total IgE was determined separately for inhalant, food, and multiple allergies, in order to verify which type of allergy is more specifically associated with high total IgE levels.

## 2. Methods

### 2.1. Patients

This retrospective study was carried out at King Abdulaziz University Hospital (KAUH), which is the referral medical center in the western region of Saudi Arabia. The electronic records of all patients who presented between January 2013 and December 2014 to the outpatient or inpatient clinics of KAUH with clinical suspicion of food or inhalant allergy were analyzed. Only patients who underwent both total IgE assay and specific allergen detection were included. Patients with no data of specific allergen testing were excluded. The protocol of this study was approved by the Biomedical Research Ethics Committee of King Abdulaziz University.

Patients were suspected for allergy based on a history of significant skin, digestive, or respiratory reaction concomitant to the exposure to any potential food or inhalant allergen. Total IgE level was determined using Unicap 100 (Pharmacia AB Diagnostics, Uppsala, Sweden). The results were collected as a continuous variable (kU/L) and the test was defined as positive for a value >195 kU/L as used in KAUH immunology laboratory. The identification of specific allergens was considered to be the golden standard and was carried out using the ImmunoCAP technology (Phadia Inc., Uppsala, Sweden). Based on the characteristics of our study population, specific allergen groups that were used in ImmunoCAP included PHAD, HX2, or MX1 in inhalant allergies and FX2, FX3, or FX5 in food allergies. For both total IgE and ImmunoCAP assays, blood samples were collected in plain tubes (without anticoagulant).

According to patient's history and clinical presentation, the population was divided into two groups: patients with suspected food allergy (group A) and those with suspected inhalant allergy (group B). A pooled analysis of the two groups was first carried out to determine the overall diagnostic value of total IgE in allergy regardless of its type. Afterwards, groups A and B were analyzed apart to determine the diagnostic value of total IgE in food and inhalant allergies, separately. In both pooled and separate analyses, subjects with positive allergen detection (positive results in ImmunoCAP) were analyzed as cases and those with negative allergen detection (negative results in ImmunoCAP) were analyzed as controls. Finally, subjects with two or more allergens identified in ImmunoCAP were compared to those with only one allergen identified, in order to assess the predictive value of total IgE in multiple allergy disorders.

### 2.2. Statistical Analysis

Statistical analysis was performed in Statistical Package for Social Sciences version 16.0 for Windows (SPSS Inc., Chicago, IL, USA). The sensitivity, specificity, positive predictive value (PPV), negative predictive value (NPV), positive likelihood ratio (+LR), and negative likelihood ratio (−LR) of total IgE level were determined in the following different clinical situations: (a) for any allergy suspected regardless of its type (groups A + B); (b) suspected food allergy (group A); (c) suspected inhalant allergy (group B); and (d) screening for multiple allergies in patients with one known allergen. Receiver operator characteristic (ROC) curves were drawn and the area under the curve (AUC) was measured to study the diagnostic value of total IgE in all four previous clinical situations. As previously specified, subjects were analyzed as cases or controls as per their results (positive or negative) in ImmunoCAP assay. Furthermore, subjects were divided into different categories as per the number of specific allergens detected and mean IgE level was compared between these categories, using independent *t*-test or one-way analysis of variance (one-way ANOVA) as appropriate. Finally, logistic regression was carried out using the presence of an allergen on the ImmunoCAP assay as the categorical variable, total IgE level as the continuous variable, and sex and age as independent variables. The statistical significance level was set at 0.05.

## 3. Results

### 3.1. Patient Characteristics

The medical records of 2641 patients were analyzed, among whom a total of 1893 (71.7%) were eligible for the study. With regard to the clinical presentation, there were 300 cases of suspicion of food allergies (group A: 60.7% males, age (mean ± SD) = 34.3 ± 20.4 years) and 1604 cases of suspected inhalant allergies (group B: 40.5% males, age (mean ± SD) = 38.5 ± 19.1 years); however, 11 patients presented twice, once with a suspicion of inhalant allergy and another time with a suspicion of food allergy, and were thus included in both groups in respective separate analysis. On the other hand, cases with ultimate positivity to both foods and inhalant allergens during the same visit were included and analyzed in their respective groups according to the clinical presentation. Patients were from a range of national backgrounds, including Saudi Arabia, other Middle East countries, Asia, and Africa (Table 1).

### 3.2. Sensitivity Analysis

In the pooled analysis (*n* = 1893), 482 (25.5%) subjects tested negative in total IgE assay (≤195 kU/L), among whom 143 were false negatives as they tested positive for either food or inhalant allergens on ImmunoCAP. Thus, total IgE assay had an overall sensitivity of 61.3%, specificity of 83.4%, PPV of 80.6%, NPV of 65.8%, +LR of 3.69, and −LR of 0.46. In separate analysis, total IgE assay had a relatively higher PPV (79.1%) in inhalant allergies than in food allergies (54.4%) and, conversely, a relatively higher (84.6%) NPV in food allergies than in inhalant allergies (67.9%). Further, the +LR and −LR values were comparable for both inhalant and food allergies (Table 2).

Comparison of means between cases and controls showed a mean total IgE level of 734.7 kU/L (*n* = 798, median = 271.0, SEM = 51.4) versus 122.1 kU/L (*n* = 806, median = 50.0, SEM = 8.9) in inhalant allergy, respectively (*p* < 0.001), and 755.2 kU/L (*n* = 77, median = 252.0, SEM = 152.7) versus 142.1 kU/L (*n* = 223, median = 52.0, SEM = 21.1) in food allergy, respectively (*p* < 0.001).

### 3.3. Receiver Operating Characteristic (ROC) Curve Analysis

**Figure 1:**
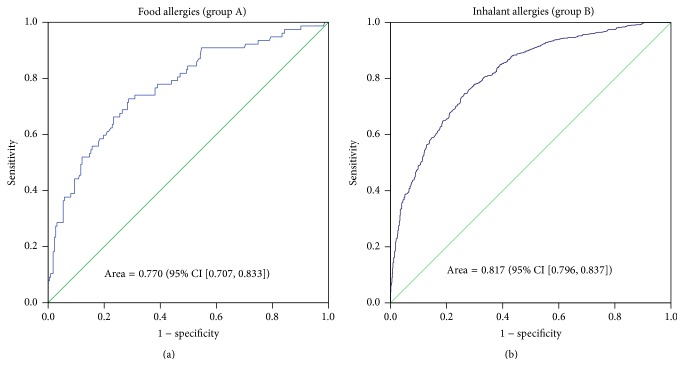
ROC curves for detecting patients with a food allergy (a) and an inhalant allergy (b) using total IgE testing. The area under the curve (AUC = 0.817, 95% CI [0.796, 0.837], *p* < 0.001) is superior in case of inhalant allergy compared to that of food allergy (AUC = 0.770, 95% CI [0.707, 0.833], *p* < 0.001).

ROC curve analysis of the diagnostic value of total IgE showed an area under the curve (AUC) = 0.770 (95% CI = 0.707–0.833, *p* < 0.001) in food allergies and AUC = 0.817 (95% CI = 0.796–0.837, *p* < 0.001) in inhalant allergies (Figure 1).

### 3.4. Sensitivity Analysis for Multiple Allergies

**Figure 2:**
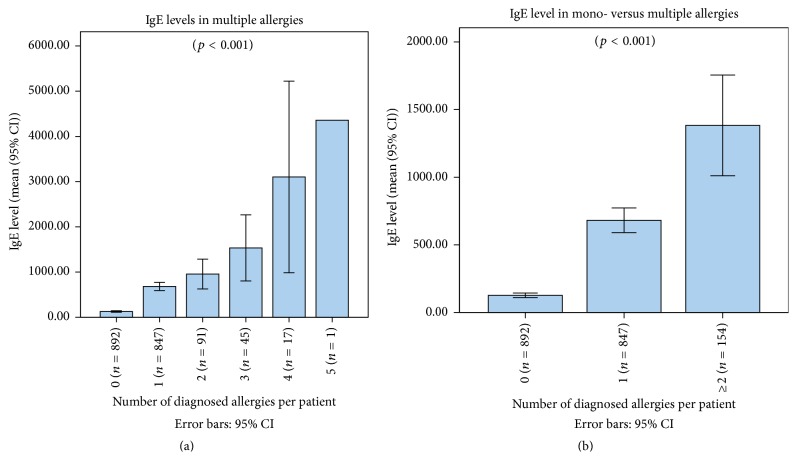
Mean (95% CI) total IgE levels in correlation with the number of allergens identified, showing (a) an increase in total IgE levels with the number of allergens (*p* < 0.001) and (b) a higher level of total IgE in cases of multiple (≥2) allergens versus one single allergen versus absence of allergen (*p* < 0.001).

One-way analysis of variance (ANOVA) showed that total IgE level significantly increased with the number of concomitant allergies (*p* < 0.001) (Figure 2).

The sensitivity of total IgE was higher (78.6%) in screening for multiple allergens in patients with an already diagnosed allergen than in the primary diagnosis of any type of allergy (61.3%), inhalant allergy (59.6%), or food allergy (55.8%). Conversely, its specificity in screening for multiple allergens is weak (41.8%), in comparison with the other diagnoses. However, a negative total IgE assay (≤195 UI/mL) predicts at 91.5% the absence of another allergen in subjects where an allergen was already detected (Table 2).

### 3.5. Receiver Operating Characteristic (ROC) Curve Analysis for Multiple Allergies

**Figure 3:**
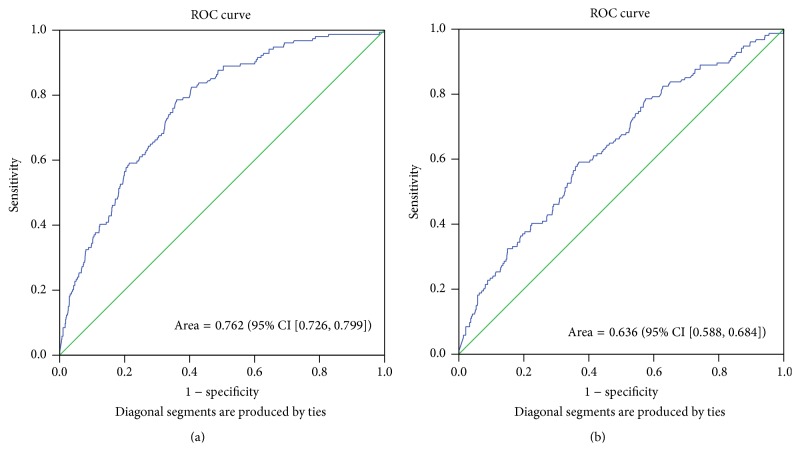
ROC curves for the diagnosis of multiple allergens in (a) patients with an unknown allergic status (AUC = 0.762 (95% CI = 0.726–0.799), *p* < 0.001) and (b) patients already known as allergic for one allergen (AUC = 0.636 (95% CI = 0.588–0.684), *p* < 0.001).

ROC curve analysis was carried out to assess the diagnostic value of total IgE in multiple allergies in two clinical situations: (a) in patients with an unknown allergic status (AUC = 0.762 (95% CI = 0.726–0.799), *p* < 0.001) and (b) in patients already known as allergic for one allergen (AUC = 0.636 (95% CI = 0.588–0.684), *p* < 0.001) (Figure 3).

### 3.6. Sensitivity of Total IgE in Diagnosing Multiple Allergies in Patients with an Already Detected Allergen, Using Different IgE Cut-Off Values

**Figure 4:**
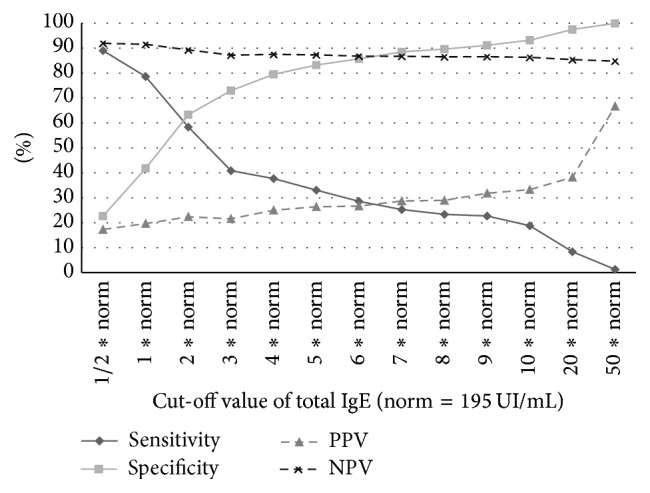
Diagnostic significance of different cut-off values of total IgE in screening for multiple allergens in patients with an already detected allergen.

Sensitivity diminishes considerably with increase in the value of total IgE used as a cut-off, while specificity increases in parallel. Further, the PPV of total IgE is weak (up to 40%) even for high cut-off values. NPV is the only constantly good diagnostic parameter (>84%), which means that a low total IgE in a patient with an already detected allergen is highly predictive of the absence of other simultaneous allergens (Figure 4).

### 3.7. Logistic Regression Analysis

Logistic regression analysis showed that age (*p* = 0.155) and sex (*p* = 0.322) were independent of the presence of an allergy and did not influence the correlation between the presence of a true allergy and the total IgE assay results. Similarly, nationality did not impact the results (*p* = 0.87), most probably because all groups except for Saudi Arabians were small in number.

## 4. Discussion

Accurate diagnosis of atopy is important for the implementation of correct management strategies [15]. Furthermore, the ability to exclude atopy as a cause of the symptoms is particularly important so that treatments with significant side effects are not used spuriously [16]. Most allergy clinicians in the Middle East still order total IgE assays for patients with suspected allergies and only proceed to specific allergy testing if the total IgE level is above a certain cut-off, which varies depending on the center. This study provides evidence that the measurement of total IgE has relatively low levels of both sensitivity and specificity. This translates into the fact that, in almost 20% of the cases, high total IgE levels do not indicate an allergy and, in up to 44% of the cases, normal levels do not necessarily indicate the absence of allergy.

Wide et al. reported the first IgE detection tool in 1967 [17], which was superseded shortly thereafter by Phadebas RAST® (radioallergosorbent test, Pharmacia Diagnostics), which measured IgE against specific allergens quantitatively. The current gold standard for assessing allergen-specific IgE in plasma or serum samples is the ImmunoCAP Specific IgE test [18]. Allergens of interest react with enzyme-labelled IgE-specific antibodies, resulting in a measurable fluorescence reaction. Such reactions can be conducted on an automated platform that enables hundreds of samples to be processed in a precise and reproducible manner. The ImmunoCAP platform is a highly sensitive and specific automated assay that is widely used worldwide for the diagnosis of allergies, but the cost and specialist technology required for the analysis mean that, in most cases, a total IgE assay is performed first as a screening tool before specific allergen tests are performed.

One of the first studies to assess the sensitivity of total IgE screening for nonspecific allergens was conducted in 2004, and it showed that screening for specific allergens was not indicated if the total IgE value was <10 kU/L [19]. However, in this study, 3 out of 73 patients with values <10 kU/L were positive for specific allergens. The cut-off used in the present study for the total IgE assay was 195 kU/L. This value was based on the protocol adopted by our biochemistry laboratory at King Abdulaziz Hospital, but no study has so far investigated this cut-off level. The cut-off is slightly higher than that published previously for IgE, notably the cut-off of 183 kU/L by Campos et al. [20] and 169 kU/L by Carosso et al. [21]. However, the normal range of total IgE can vary between ethnic groups, and those in the Middle East tend to have higher levels of circulating IgE than western populations [21, 22]. In this study, we could not determine the influence of ethnicity as the majority of the population was from Saudi Arabia, and the nationality cannot accurately indicate the ethnicity. In addition to the influence of ethnicity, total IgE levels vary depending on geographic area [23], smoking [21], and age [24]. Sex was also reported to be an influencing factor, with higher mean levels of total IgE found in a cohort of boys compared to girls in a study from South Korea [25]. However, we found no difference in cross gender comparison of means in total IgE levels nor in logistic regression. Moreover, we did not find age to have a significant influence on the IgE levels.

In this study, when we analyzed food and inhalant allergies separately, we found that the total IgE level had a higher PPV for inhalant allergies (79.1%) than for food allergies (54.4%), while it had a higher NPV for food allergies (84.6%) than for inhalant allergies (67.9%). Further, the diagnostic value was relatively higher for inhalant allergies than for food allergies in ROC curves, but we cannot conclude to the efficacy of use of total IgE as a diagnostic test for inhalant allergies.

We also determined the specificity and sensitivity of the total IgE level for the diagnosis of multiple allergies. The sensitivity of total IgE (cut-off >195 UI) in diagnosing multiple allergens was higher than that in the primary diagnosis of any type of allergy. Expectedly, when different values of total IgE levels were analyzed as cut-offs in the diagnosis of multiple allergies in patients with a known allergy, we found that sensitivity decreases for higher values and specificity decreases for lower values. Practically, in patients with a documented allergen, even very high levels of total IgE are a poor indicator of the existence of another allergen. However, in case of identification of multiple allergens, total IgE showed efficacy in monitoring the efficacy of polydesensitisation methods, such as IFN-gamma therapy, where a decrease in total IgE levels significantly indicated an improvement in the polysensitised status [26].

Further, our study showed that NPV of total IgE (91.5%) was best in the diagnosis of multiple allergies, which means that a low total IgE level in a patient with one known allergen is highly predictive of the absence of a second allergen. Thus, total IgE could be helpful to rule out a polysensitisation syndrome in patients where a specific allergen has already been diagnosed. This can be especially indicated when the clinical context is not conclusive enough to guide further specific allergens identification. However, such conclusions require stronger evidence.

## 5. Limitations

As this was a retrospective design, many confounders could not be collected and analyzed, such as smoking that could have been a major confounder for increased serum total IgE levels [21, 24]. A prospective controlled trial that assesses the outcomes after randomized patients are assigned to undergo total IgE testing or not is now warranted.

## 6. Conclusion

The results of this study indicate that total IgE assay is not efficient as a diagnostic test for allergy diagnosis in Saudi patients, especially in food allergies. The best diagnostic strategy in allergology should refer to the proper selection and interpretation of specific IgE testing, sustained by a comprehensive atopic history of the patient. Nevertheless, total IgE could be proposed in ruling out multiple allergic disorders in case of one specific allergen identification in patients with nonconclusive history.

## Figures and Tables

**Table 1:** Population characteristics.

Parameter		Inhalant allergy (*n* = 1604)		Food allergy (*n* = 300)	
		Frequency/mean	%/SD	Frequency/mean	%/SD
Gender	Male	650	40.5	118	60.7
	Female	954	59.5	182	39.3

Age		38.5	19.1	34.3	20.4

Nationality	Arabia	1222	76.2	196	65.3
	Middle East	134	8.4	32	10.7
	Asia	142	8.9	51	17.0
	Africa	91	5.6	16	5.3
	Others	15	0.9	5	1.7

IgE level		426.9	1083.3	299.45	775.78

**Table 2:** Diagnostic value of total IgE (positive if >195 IU/mL) in food, inhalant, and multiple allergies.

Parameter	Food allergy (*n* = 300)		Inhalant allergy (*n* = 1604)		Multiple allergies (*n* = 1893)		All allergy types (*n* = 1893)	
Sensitivity	55.8%	*p* < 0.001	59.6%	*p* < 0.001	78.6%	*p* < 0.001	61.3%	*p* < 0.001
Specificity	83.9%		84.4%		41.8%		83.4%	
PPV	54.4%		79.1%		19.7%		80.6%	
NPV	84.6%		67.9%		91.5%		65.8%	
+LR	3.820		3.465		1.35		3.692	
−LR	0.479		0.527		0.51		0.464	

PPV: positive predictive value; NPV: negative predictive value; +LR: positive likelihood ratio; −LR: negative likelihood ratio.
